# Uterine Vasculature Remodeling in Human Pregnancy Involves Functional Macrochimerism by Endothelial Colony Forming Cells of Fetal Origin

**DOI:** 10.1002/stem.1385

**Published:** 2013-07-05

**Authors:** Peter I Sipos, Willem Rens, HÉlène Schlecht, Xiaohu Fan, Mark Wareing, Christina Hayward, Carl A Hubel, Stephane Bourque, Philip N Baker, Sandra T Davidge, Colin P Sibley, Ian P Crocker

**Affiliations:** a Maternal and Fetal Health Research Centre, University of Manchester, Manchester Academic Health Science CentreManchester, United Kingdom; b Department of Veterinary Medicine, Cambridge Veterinary School, University of CambridgeCambridge, United Kingdom; c National Genetics Reference Laboratory (Manchester), St Mary’s HospitalManchester, United Kingdom; d Department of Pediatrics and Pharmacology, University of AlbertaEdmonton, Alberta, Canada; e Magee-Women’s Research Institute and Department Obstetrics, Gynecology & Reproductive Sciences, University of PittsburghPittsburgh, Pennsylvania, USA; f Women and Children’s Health Research Institute, University of AlbertaEdmonton, Alberta, Canada

**Keywords:** Neovascularization, Physiologic, Maternal-Fetal Exchange, Chimerism, Circulation, Uteroplacenta

## Abstract

The potency of adult-derived circulating progenitor endothelial colony forming cells (ECFCs) is drastically surpassed by their fetal counterparts. Human pregnancy is associated with robust intensification of blood flow and vascular expansion in the uterus, crucial for placental perfusion and fetal supply. Here, we investigate whether fetal ECFCs transmigrate to maternal bloodstream and home to locations of maternal vasculogenesis, primarily the pregnant uterus. In the first instance, endothelial-like cells, originating from mouse fetuses expressing paternal *eGFP*, were identified within uterine endothelia. Subsequently, LacZ or enhanced green fluorescent protein (eGFP)-labeled human fetal ECFCs, transplanted into immunodeficient (NOD/SCID) fetuses on D15.5 pregnancy, showed similar integration into the mouse uterus by term. Mature endothelial controls (human umbilical vein endothelial cells), similarly introduced, were unequivocally absent. In humans, *SRY* was detected in 6 of 12 myometrial microvessels obtained from women delivering male babies. The copy number was calculated at 175 [IQR 149–471] fetal cells per millimeter square endothelium, constituting 12.5% of maternal vessel lumina. Cross-sections of similar human vessels, hybridized for Y-chromosome, positively identified endothelial-associated fetal cells. It appears that through ECFC donation, fetuses assist maternal uterine vascular expansion in pregnancy, potentiating placental perfusion and consequently their own fetal supply. In addition to fetal growth, this cellular mechanism holds implications for materno-fetal immune interactions and long-term maternal vascular health.

## INTRODUCTION

Pre-eclampsia and intrauterine growth restriction (IUGR) are late pregnancy complications associated with insufficient uterine vascular density and suboptimal placental perfusion. These pathologies affect up to 5% and 10% of pregnancies, respectively [1–3] and have immediate and long-term health implications for mother and fetus [4, 5]. The cellular source of optimal vascular development within the pregnant uterus is unexplored. Human vascular development relies upon endothelial expansion achieved by angiogenesis, remodeling of mature endothelial cells, and by progenitor-driven types of vessel formation, that is, vascularization and vasculogenesis, distinguished by the presence or absence of pre-existent vessels. For angiogenesis, vessel formation is limited by the nonproliferative nature of mature endothelial cells. This mechanism may be insufficient for the exaggerated vascular expansion of pregnancy.

For vasculogenesis and vascularization, vessel expansion is boosted by highly proliferative endothelial progenitor cells (EPCs). From the many putative EPCs, endothelial colony forming cells (ECFCs) are of classic endothelial and true progenitor characteristics [6]. These cells are actively recruited in humans from the bone marrow [7], and we have confirmed their physiological vasculogenic capabilities in vivo [8]. Putative EPCs have a suggested, but unconfirmed role for physiological endometrial angiogenesis in the mouse and human [9, 10]. Thus, the participation of ECFCs in the pregnant uterus would likewise be anticipated.

In the maternal circulation, most studies report an increase in ECFC number (or their equivalents) through the second half of pregnancy [11], with cells significantly elevated in the last trimester [12]. Whether this reflects increased production or decreased consumption hampers interpretation. As the rate of fetal development declines, functional capacities of fetal ECFCs decrease with gestational age [13]. However, the course of changes in the number of fetal ECFCs is debated [14, 15].

The clonogenic classification of ECFCs by Ingram et al. [16] defined three subsets of cells, with varying proliferative capacity. Of these, the most efficient, high proliferative potential ECFCs have high telomerase activity and can duplicate for more than a hundred times. It is of note that these cells are exclusive to the fetus and absent in the adult [16, 17]. Moreover, there is greater abundance in general of EPCs in the fetal circulation [18]. With our previous results showing an age-related decline in function, as specified by 6-*o*-sulfation of heparan sulfate proteoglycans [19], these observations portend that adult EPCs fall short of their fetal counterparts, both numerically and functionally, regarding vessel formation and vascular repair.

Throughout human pregnancy the uterus undergoes extensive adaptations to accommodate the growing fetus [20]. Perhaps the most striking is the rapid expansion of the uterine microvasculature [21], supporting an increase in uterine blood flow from 20 to 50 mL/minute in the nonpregnant state, to around 900 mL/minute at term [22]. To accommodate, uterine spiral arteries undergo extensive remodeling by placental-derived extravillous trophoblasts (EVT). These invasive cells interact with the arterial smooth muscle and endothelium, transforming vessels into wide-bore, high-flow conduits [23, 24]. Although originally conceived that EVT trans-differentiated to replace this liberated endothelia [25–28], recent evidence supports a practice of re-endothelization within these spiral arteries [29] and an active process of de novo endothelial replacement.

We have previously demonstrated active migration and uptake of ECFCs from the fetus to the placenta and shown their role in normal placental vasculogenesis [8]. Here we considered whether these cells, with their superior potency, traverse the placenta, circulate in the maternal blood and home to sites of intensive vessel formation, including the uterine microvasculature. Although fetal cells are found in maternal blood and can survive in a range of tissues after pregnancy [30], true confirmation of chimeric EPCs (including ECFCs) in the maternal circulation is lacking. To investigate, we have (a) tracked genetically labeled cells of the mouse fetus, ubiquitously expressing enhanced green fluorescent protein (eGFP), to the maternal uterine endothelium; (b) introduced culture-derived human fetal ECFCs into the circulation of Non-Obese Diabetes/Severe Combined Immunodeficient (NOD/SCID) fetuses during intact mouse pregnancy, confirming the subsequent integration into the maternal uterus, and (c) identified male cells within microvessels of human uterine myometrial biopsies, estimating their relative abundance by reverse transcription-quantitative polymerase chain reaction (RT-QPCR) and confirming their localization within vessel walls by fluorescence in situ hybridization (FISH).

## METHODS

### Ethical Approval

All patients gave informed consent for participation, and the study was approved by the North West Research Ethics Committee, U.K. and the Ethics Committee of Central Manchester Foundation Trust Hospitals, U.K. All protocols related to animal experiments were approved by the University of Alberta Health Sciences Animal Policy and Welfare Committee in accordance with the Canadian Council on Animal Care guidelines.

### Transmigratory Model of Fetal Cells to the Mouse Uterus

Virgin wild-type C57 female mice (C57BL/6NJ, Jackson Lab, Sacramento, CA, http://www.jacksonimmuno.com) were mated with transgenic male mice, heterozygous to the *EGFP* gene, under the control of a chicken β-actin promoter and cytomegalovirus enhancer (CByJ.B6-Tg(CAG-EGFP)1Osb/J, Jackson Lab). Prior to mating, eGFP was absent in the maternal organism. After mating, a portion of generated pups were shown to ubiquitously express eGFP as a result of paternal inheritance. Using an optical imager (Olympus, Tokyo, Japan, http://www.olympus-global.com), eGFP expressing cells of fetal origin were localized within maternal uterus and its arterial system. Cross-sections of tissue (10 μm) were immunofluorescently stained, using standard protocols [31] for von Willebrand factor (Millipore, Billerica, MA, http://www.millipore.com), an endothelial cell marker.

### Culture Expansion of ECFCs

ECFC were expanded from fresh cord blood as previously described by Mead et al. [18]. Rat-tail collagen-I coated plates (BD, Oxford, U.K., http://www.bd.com) and Endothelial Growth Medium (EGM-2) Lonza, Slough, U.K., http://www.lonza.com were used.

### Lentiviral Vector Transduction of ECFCs

A vesicular stomatitis virus-pseudotyped lentiviral vector harboring eGFP under the ubiquitous EF1α promoter and a lentiviral vector, harboring β-galactosidase (LacZ) under the CMV promoter were used: pLenti6/V5-GW/lacZ from ViraPower (Invitrogen, Carlsbad, CA, http://www.invitrogen.com). For packaging, 293T cells were cotransfected with pDelta 8.74 and pMD2G plasmid using polyethyleneimine. For transduction of ECFCs, 1 × 10^7^ cells were incubated overnight with 2 × 10^7^ Transducing Units of lentiviral vector, in the presence protamine sulfate (7 μg/mL).

### Characterization of Fetal ECFC

Using a FACSAria (BD, Toronto, CA), unlabeled ECFCs, eGFP-ECFCs, human umbilical vein endothelial cells (HUVECs), and CMFDA-HUVECs (HUVECs labeled with the green Cell Tracker CMFDA in accordance with manufacturer’s instructions (Molecular Probes Cell, Invitrogen Life Technologies, Paisley, U.K. http://www.invitrogen.com) were single cell sorted into 96-well culture plates, precoated with collagen-I (BD) (ECFCs) or 1% gelatin (HUVECs). Further cultures were prepared from seeding densities of 5,000 cells per centimeter square. ECFCs were cultured in EGM-2 media (Lonza Vervieres, S.p.r.l. Verviers, Belgium), supplemented with 1.5% (v/v) amphotericin-B, 1% (v/v) penicillin/streptomycin, and 0.15% (v/v) gentamycin, and HUVECs were grown in Dulbecco’s modified Eagle’s medium (DMEM) with similar supplements and 20% (v/v) fetal bovine serum.

To measure in vivo angiogenic capacity, ECFCs mixed with adipose-derived stem cells (ADSCs) at 4:1 were suspended in a collagen-fibronectin matrix [18]. Contracted gels were implanted subcutaneously into the flanks of NOD/SCID immunodeficient mice (Strain 005557, Jackson Laboratory) under isoflurane anesthesia and mice were allowed to recover for 14 days, with continuous amoxicillin/clavulanate prophylaxis. Harvested implants were examined under optical imager and fluorescent microscope.

### Mouse Transplantation of Human ECFCs into Immunocompromised Fetuses

On D15.5 pregnancy, as determined by plug test, NOD/SCID mice under isoflurane anesthetic were monitored on heating pads while abdominal fur was removed and skin disinfected. Pregnancy and fetal position were confirmed using a VS 40 high frequency, high-resolution ultrasound platform with a 30 MHz probe (VisualSonic, Toronto, Canada, http://www.visualsonics.com) (Supporting Information Video 1).

Live fetuses, in an optimal position with their abdomens facing the maternal abdominal wall, were selected for intervention (Supporting Information Video 1a). Hearts were centered for needle insertion. 1 × 10^7^ EGFP-ECFCs, LacZ-ECFCs, or CMFDA-HUVECs diluted in 70 mL supplemented EGM-2 or DMEM were taken into a 250 mL GasTight luer type syringe (Hamilton AG, Bonaduz, Switzerland, http://www.hamiltoncompany.com) with 32G × 12 mm needle. By ultrasonic guidance, the needle was inserted sequentially through the maternal skin, abdominal and uterine walls into the amniotic cavity and fetal heart. After piercing the thoracic skin, the tip was guided into the cardiac cavity (Supporting Information Video 1b). Here the syringe was injected for more than 4–5 seconds and gently withdrawn (Supporting Information Video 1c). The fetus was monitored for several minutes to confirm a reestablished heart rate and to observe potential bleeding within the amniotic cavity. As the syringe routinely retains 35 mL in the luer, the amount of injected cells was estimated at 5 × 10^6^ per fetus. With transplantation complete, the mother was allowed to recover and the pregnancy to continue for a further 3 days, continually receiving prophylactic antibiotics (amoxicillin/clavulanate in the drinking water). The maternal uterus and broad ligaments were subsequently examined with optical imager (Olympus OV-100) and dissected placentas bisected. One half were fixed in Z-FIX (Anatech, Battle Creek, USA, http://www.anatechltdusa.com), the other cryopreserved and sectioned for histology and immunohistochemistry, highlighting Claudin 5 and Connexin 40, components of endothelial tight and gap junctions, respectively.

### RT-QPCR of Maternal Uterine Microvessels

Human microvessels, approximately 100 μm diameter and 6 mm length, were isolated from muscular tissue biopsies taken at caesarean section from the upper uterine segment. Pregnancies with male babies were primarily considered, however, microvessels from women with female infants and placentas of newborn males were also used as external negative and positive controls, respectively. DNA was extracted using the Qiagen (Valencia, CA, http://www1.qiagen.com) DNA extraction kit according to manufacturer’s instructions. DNA concentration and purity were determined by Nanodrop ND-1000 (Thermo Scientific, Wilmington, USA, http://www.nanodrop.com) using spectrophotometric absorbance at 260 nm and absorbance ratio at 260/280 nm, respectively. After normalization of the DNA concentration (2 ng/μL, DNAse-free water), samples underwent RT-QPCR. The male-specific *SRY* gene (eight replicates) and, for internal control, the endogenous *CCR5* gene (two replicates) were amplified (*SRY* and *CCR5* assay (20×), TaqMan Universal PCR Master Mix, No AmpErase UNG, ABI Prism7900 Sequence Detection System [Applied Biosystems, Carlsbad, CA, http://www.appliedbiosystems.com]). PCR settings were: 50°C 2 minutes, 95°C 10 minutes, 45 cycles 95°C 15 seconds, 60°C 1 minute [32]. Experiments were considered successful if all samples had cycle thresholds (ct) <40 for *CCR5*, while negative controls had ≥45 and positive controls <38 ct values for *SRY*. Test samples were considered positive for the presence of *SRY* in the event of at least one replicate having repeated ct value <40 in two independent experiments. Standard curves were generated using human male genomic DNA (20 −0.02 ng/μL, Promega, Southampton, U.K. http://www.promega.com) and resultant ct values were used to determine male cells per vessel extract (ABI SDS software). This technique, in our hands, has a specificity of 100% and 98% sensitivity [32].

### Sex Chromosome FISH

Similar vessels were used as for RT-QPCR above. Samples, initially embedded in OCT, were first sectioned (10 μm thick) and then fixed with 4% (w/v) paraformaldehyde. Slides were baked at 64°C, tissues were permeabilized by a sequence of citrate buffer treatment, immersion into saponin (1.5% (w/v)) and Triton-X (1.5% (w/v)) with added glycerol (20% (v/v)), followed by five freeze-thaw cycles in liquid nitrogen and glycerol (20% (v/v)). The process of permeabilization was completed by immersion into pepsin 1% (w/v) in 10 mM HCl. DNA was denatured at 72°C and slides were incubated with denatured Cy-3-labeled Y-chromosome-specific whole-chromosome paints, and fluorescein isothiocyanate(FITC)-labeled chromosome 20 or 15 equivalents (StarFISH, Cambio Ltd, Dry Drayton, U.K., http://www.cambio.co.uk). Slides were counterstained with 4’,6-diamidino-2-phenylindole (DAPI) and analyzed using a cytogenetics workstation (Leica, Peterborough, U.K., http://www.leica.com). Chromosomal signals were determined as intranuclear fluorescent events within the focal plane of nuclei, having identical shape, size, and calculated fluorescent brightness equal to male placental controls.

## RESULTS

### Fetal Endothelial-like Cells Found in the Maternal Uterine Vessels of the Mouse

**Figure 1:**
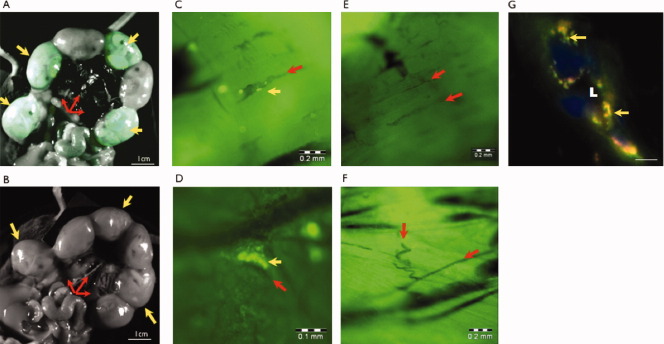
Fetal cells of endothelial characteristics cross the mouse placenta and colonize the uterine microvasculature in a transgenic murine model of enhanced green fluorescent protein (eGFP)-expressing offspring. Images taken with small animal imager demonstrated eGFP-positive offspring (yellow arrows) in dissected intact uterine horns of eGFP-negative mothers, supplied by vessels of broad ligaments (red arrows, A). Pups (yellow arrows) did not express *eGFP* in the event of control matings (B). Fluorescent cells of fetal origin were detected by small animal imager at D18.5 within maternal uteri (C) and broad ligaments (D). These cells (yellow arrows) were aligned along lumina of arterial and venous branches (red arrows), and frequently formed obvious vascular colonies, while eGFP signals were undetected in uteri (E) and broad ligaments (F) of controls. With crossmating, immunofluorescence of uterine cross-sections (G) showed coexpression (amber, yellow arrows) of fetus-associated eGFP (green) and endothelial Von Willebrand Factor (red) in cells surrounding the vessel lumina (L), confirming fetal origin and endothelial character. Blue = Nuclei. Scale bars (unless indicated) = 10 μm.

Native virgin female mice, mated with transgenic males heterozygous for *eGFP*, resulted in litters of eGFP-positive and −negative pups (Fig. 1A). In control experiments, all pups derived from mating native mice were homozygous eGFP-negative (Fig. 1B). Using optical imager at D18.5, fluorescent cells, derived from eGFP-expressing fetuses, were observed within uteri of pregnant dams (*n* = 6, Fig. 1C) and also in their broad ligaments, a peritoneal fold, which includes the vascular supply of the uterus (Fig. 1D). These cells were found distal to placental implantation sites, and thus potential regions of EVT invasion. Close inspection (10–16× magnification) showed fluorescent fetal cells lining-up in colonies subjacent to vascular lumina. Video recordings showed these chimeric cells to be unaffected by circulating erythrocytes, suggesting complete vascular infiltration (Supporting Information Video 2). In controls, no fluorescent signals resembling uterine or broad ligament colonizations were detected (*n* = 3; Fig. 1E, 1F, respectively). Dual fluorescence from intrinsic eGFP-expression and staining for Von Willebrand Factor, a recognized endothelial marker, confirmed endothelial lineage for these transmigrated fetal cells (Fig. 1G). These observations, demonstrating fetal-derived cells of endothelial progeny (but not unequivocally ECFCs) within the murine uterus, prompted use of a further model designed more specifically to investigate human ECFC behavior.

### Human Fetal ECFCs Transmigrate the Murine Placenta and Exhibit Vasculogenic Function

Human fetal ECFCs, isolated from neonatal umbilical cord blood using established culture techniques [18], were phenotyped for authenticity before transfection by retroviral gene delivery systems transducing the *eGFP* or *LacZ* reporter genes for ubiquitous labeling (eGFP-ECFCs or LacZ-ECFCs). They were found to express CD31, CD105, CD146, CD34, VEGFR-2, stain with UEA-lectin, take up Ac-LDL, and form tubes on Matrigel, suggestive of endothelial lineage. They did not express CD14, CD45, or CD133, excluding mononuclear hematopoietic origin. Both untreated and modified cells proliferated in single cell cultures and showed optimal and unaffected angiogenic capabilities when subcutaneously implanted in ADSC, collagen and fibronectin combined artificial tissue blocks into immunodeficient NOD/SCID mice, two features unique to ECFCs and essential for their full characterization [6]. The expression of reporter genes was confirmed in these fetal ECFCs before subsequent use for transplantation.

**Figure 2:**
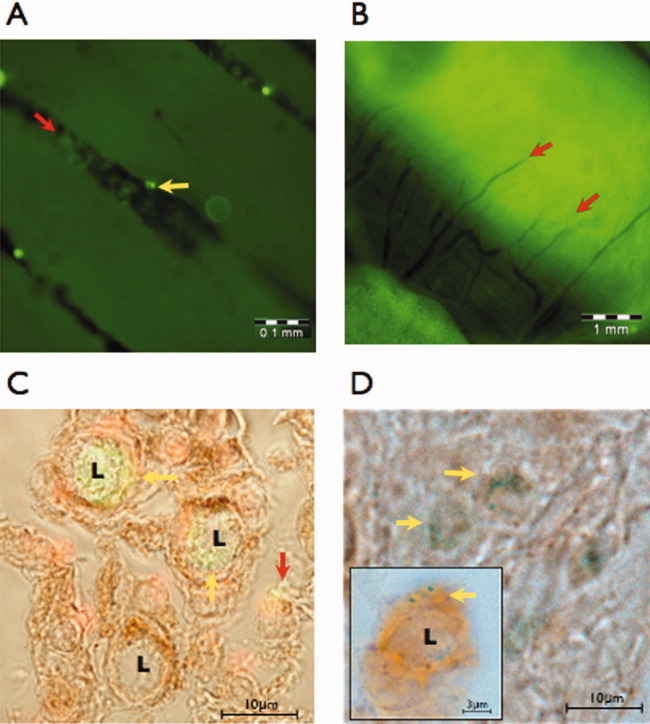
Transplanted fetal-derived human endothelial colony forming cells (ECFCs) traverse the mouse placenta and home to the pregnant uterus. On D15.5, enhanced green fluorescent protein (eGFP) and LacZ expressing culture-propagated human cord blood ECFCs were transplanted by ultrasound guided intracardiac injection into Non-Obese Diabetes/Severe Combined Immunodeficient (NOD/SCID) mouse fetuses. Using a small animal imager, eGFP-fluorescent cells (yellow arrows) were subsequently located along the vascular lumina (red arrows) within the pregnant mouse uterus (A). Although detectable in the blood of mouse placentas [8], fluorescent-tracked mature endothelial cells (human umbilical vein endothelial cells) could not be defined in the maternal uterine microvasculature (B). Combined fluorescence and light microscopy of uterine tissues (red nuclei) showed transplanted eGFP-ECFCs (yellow arrows) undertaking de novo lumen (L) formation. Positive staining for claudin-5, a tight junction marker (brown stain), confirms their integration, while nonintegrated ECFCs fail to express tight junctions (red arrow). (C) Transplanted human LacZ-ECFCs (punctate blue stain, yellow arrows) similarly demonstrated vasculogenic activities, including vacuole formation (L, insert), and expressed the gap junction marker connexin 40 (brown), confirming full vascular integration (D).

In pregnant NOD/SCID mice, identical cells were intracardiac injected under sonographic guidance into fetuses at D15.5 (Supporting Information Video 1). After 3.5 days incubation, human eGFP-ECFCs (*n* = 7 mouse pregnancies) or LacZ-ECFCs (*n* = 5) were tracked to the maternal mouse uteri on D18.5. Within these uteri, groups of labeled cells were observed associating with arterial walls (Fig. 2A). Real-time video (Supporting Information Video 3) demonstrated their retention within vessels, despite visible and contiguous blood flow. Parallel studies, with injected eGFP-HUVEC, CMFDA-HUVEC, or EGM-2 medium alone, failed to demonstrate evidence of transmigration and uterine relocation of fluorescent cells (Fig. 2B). Tissue staining for tight and gap junction markers, claudin 5 and connexin 40, confirmed mural integration of eGFP/LacZ-ECFCs (Fig. 2C, 2D, respectively). Graft cells were observed forming complex vascular structures (Fig. 2C) and de novo lumina by vacuolization (Fig. 2D), providing combined evidence for cross-species capacity for vasculogenesis and vascularization.

### Fetal Derived Endothelial Cells Located in the Human Uterine Vasculature

**Figure 3:**
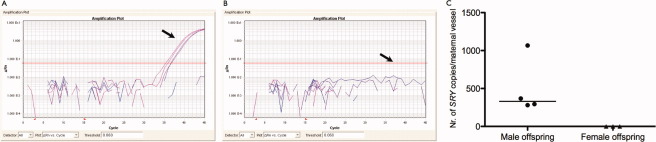
The presence of fetal-derived male cells within maternal human uterine vessels. Reverse transcription quantitative polymerase chain reaction (PCR) amplification plots of the male-specific *SRY* gene in microvessels from biopsies of the upper uterine segments showed gene presence in 6 of 12 cases of mothers with male offspring (A, arrow points at positive *SRY-*amplification, fluorescence higher than threshold [red line]). Gene copies were absent in women with female babies (B, *n* = 3, arrow points at intact detection threshold). Horizontal axes: PCR cycle numbers, vertical axes: Δ*R*n (arbitrary units (log)). In 4 of 6 positive cases the copies of SRY were successfully quantified (C).

For human observations, microarteries (60–140 μm internal diameter × 4–8 mm length) were dissected from uterine muscle biopsies obtained from elective caesarean section of 12 uncomplicated pregnancies with singleton male babies. The presence of male specific *SRY* gene and Y chromosome were explored by RT-QPCR and FISH, respectively. The presence of *SRY* of fetal origin was detected in the extracted DNA of these vessels in at least one in eight replicate reactions in two independent sets of experiments, Overall, the *SRY* gene was detected in 6 of 12 pregnant women (Fig. 3A). Uterine microvessels from mothers with female babies were used as external negative controls (Fig. 3B, *n* = 3) and placental chorionic plate arteries of a male baby for positive confirmation. Using a dilution curve and the total quantity of DNA extracted, the *SRY* copies in positive vessels were successfully quantified in 4 of 6 positive samples as 331 [IQR 283–892] (Fig. 3C). Using an estimated length (6 mm) and diameter of vessel (100 μm) a crude calculation yields an internal surface area of 1.89 mm^2^, giving 175 [IQR 149–471] fetal cells per millimeter square endothelium. Assuming a single endothelial cell has a diameter of 30 μm, 1 mm^2^ vascular lining is covered by 1,411 endothelial cells in total. The proportion of fetal-derived cells within the pregnant uterus, therefore, approximates to 12.4% of the total microvascular endothelium at term.

**Figure 4:**
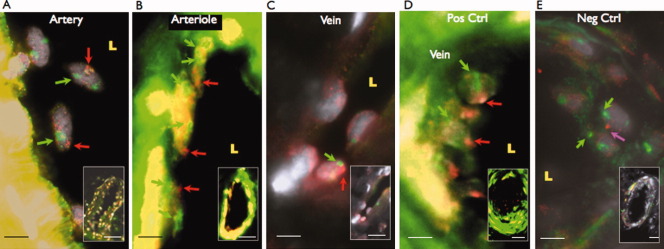
Endothelial location of fetal-derived cells within human uterine vessels. Y-chromosome-specific FISH in uterine tissue cross-sections detected male cells in the endothelium in 8 of 11 mothers with male babies. Examples given for prominent Y-signals in detached endothelial cells of a larger arterial branch (A), a colony of fetal cells in the intact endothelial lining of an arteriole (B) and fetal cells in a venous endothelium (C). Male placentas were used as positive controls (D, *n* = 4), and uterine tissue of mothers with female offspring as negatives (E, *n* = 2, magenta arrow = artifact resembling Y-signal but outside nuclear margin). Somatic chromosomes were hybridized for internal control (red color/arrows: Cy3-conjugated Y chromosomes, green color/arrows: Fluorescein isothiocyanate (FITC)-conjugated chromosomes 20 or 15, gray: 4’,6-diamidino-2-phenylindole (DAPI), L: lumen, Scale bars = 5 μm, 20 μm (insets), 50 μm (inset A)).

To further assess location of these fetal cells within anatomical structures of these vessels, tissue cross-sections were hybridized in situ for Y-chromosome simultaneously with chromosomes 20 or 15 as internal controls. Y-chromosome-containing fetal cells were detected in at least one of three sections of uterine microvessels from 8 of 11 pregnancies studied (Fig. 4A–4C). Individual fetal cells and colonies were observed in the endothelium of larger arteries (Fig. 4A), arterioles (Fig. 4B), and veins (Fig. 4C). Detachment of endothelial cells from the basal membrane allowed superior chromosome detection within the elongated nuclei (Fig. 4A). Fetal cells were not observed in nonendothelial vascular compartments, although recognition in the arterial adventitia was difficult, given its high autofluorescence. Placental chorionic plate arteries, associated with male neonates (*n* = 4) were used as confirmatory positives (Fig. 4D), while uterine biopsies from pregnancies eventuating female offspring (*n* = 2) were used as negative controls (Fig. 4E). Although the incidence of positive findings were lower than corresponding RT-QPCR, this discrepancy may be explained by technical variables, most notably poor penetration of FISH probes within the myometrial tissue. To ensure specificity, chromosomal signals weaker than controls were invariably disregarded, admittedly at the cost of overlooking fetal cells.

## DISCUSSION

From initial transgenic matings, eGFP-expressing cells of fetal origin were identified as resident within the pregnant mouse uterus. These cells, of confirmed endothelial lineage, showed positive transmigratory capacity, in line with past maternal models of mouse vascular injury [33]. However, ECFCs are solely distinguished from mature endothelial cells by their functional characteristics [34]. It was, therefore, imperative to combine our search for fetal-derived cells in the uterus, with a mouse model of chimerism incorporating functional human ECFCs. The further tracking of fetal-derived human ECFCs, cardiac-injected within the fetus, showed equal infiltration of the pregnant mouse uterus, with additional de novo vessel formation. This transmigration and vascularization was peculiar to fetal ECFCs and absent with mature endothelial cells (HUVECs), similarly transplanted.

Although the mechanism by which fetal ECFCs migrated across the feto-maternal barrier and integrated the maternal uterus was undefined in this study. For progression of the cells, a passive, accidental movement cannot be excluded, as elicited by hemodynamic disturbances within the placental intervillous spaces [35]. Chemotaxis to agents which stimulate ECFC passage may be another mechanism, as a number of confirmed chemotactic agents, EMAP-2, SDF-1, IL-8, vascular endothelial growth factor (VEGF), and Gro-α, are reported in both uterine and placental tissues [36–40]. For transmigrating the feto-maternal barrier, their previous characterization proposes a matrix metalloproteinase-2 (MMP-2)-dependent process, for which these cells are highly regarded [41]. Although HUVECs also respond to these agents, and produce MMPs, their migratory capacities and expression of MMP-2 and collagen binding receptor CD44 are inferior [42, 43]. Irrespective of placental integration, ECFC survival is favored in the hostile environment of maternal immunity, as these cells are not allogenic, they do not express major histocompatibility complex II (MHC-II) and their level of MHC-I is minimal, even after Interferon-gamma-stimulation [44].

The further dominance of ECFCs over HUVECs is also apparent at the time of vessel invasion. ECFC integration depends upon active urokinase-like plasminogen activator receptor (uPAR), preferentially found in cellular caveolae [45]. uPAR and caveolae are both upregulated by VEGF [45, 46]. With ECFCs in general having higher expression of uPA, uPAR, and VEGFR-2, along with greater MMP-2 activity than HUVECs [42, 46, 47], ECFCs again hold the clear advantage. For de novo vasculogenesis, ECFCs actively excrete components of the vascular basal membrane and extracellular matrix. Differentiated endothelial cells can also produce laminin, collagen and fibronectin, but they fail to deposit these agents extracellularly [48]. Lastly, their restricted proliferation, inability to form colonies and incapacity for integration (Sipos, 2011 #293), would limit maternal vasculogenesis by HUVEC. The fact HUVECs were absent in their entirety in the uterine tissues of both mouse and humans in these studies, and that they failed to integrate into placental vessels in our past transplantation studies where ECFCs succeeded ([8]), strongly implies that mature endothelial cells, unlike fetal ECFCs, fail at the first hurdle in placental transmigration, unable to infiltrate placental vessels, let alone traverse the placenta for eventual uterine residency.

Within our studies, the incorporation of human fetal ECFCs in the mouse uterus, although patchy, must be taken in the context of the in vivo period of incubation, that is, 3.5 days, as compared to 280 days of human pregnancy. Given that these fetal ECFCs can proliferate for at least 200 days in culture without signs of senescence, a single transmigratory cell could yield 1,023 daughter cells within second and third trimesters [16]. Considering this, and also the observed absence of fetal-introduced HUVECs in the maternal vasculature, it is perhaps not surprising that the frequency of fetal cells within human uterine vessels was as high as 12.4% at term, as these cells are likely ECFCs and not mature endothelial cell equivalents. This level of mass migration, or in situ expansion, supersedes that of microchimerism reported in other maternal tissues [49, 50]. This frequency also hints at a physiological role in the human and cannot be explained by coincidental inoculation.

Beyond the previously mentioned low MHC expression of ECFCs and endothelial cells derived from them, it is reasonable to believe that upon full maturation of subsequent generations of daughter cells, MHC expression may notably increase over time. Within the maternal circulation and entrenched within the uterus, it is difficult to envisage how these daughter cells avoid immune detection, or instigate maternal tolerance. Nonetheless, this same conundrum befits the fetus, and more specifically the placenta, and although hotly debated, appears to have basis in unresponsive T-lymphocytes, with a direct requirement for regulatory T-cells [51]. Perhaps the most important question is how this acceptance can be maintained in a past-pregnant woman, sometimes decades post-delivery. There is no doubt that after normal pregnancy, or pregnancy loss, fetal cells remain distributed widely in maternal tissues, differentiated into numerous cell phenotypes. Murine models of persistent microchimerism demonstrate clonal deletion in the thymus of self-reactive T-cells which could prolong this transitory tolerance [52]. Nevertheless, cytotoxic T-cells, specific for fetal cells, can be continually isolated in women post-delivery [53], suggesting a more delicate balance between tolerance and active immunity. The maternal impact of protracted microchimerism is also undecided. It is speculated that harboring fetal cells could benefit or disadvantage maternal health and future pregnancies, contributing to tissue repair and tumor surveillance on one-hand or encouraging autoimmune disease and secondary miscarriage on the other.

By producing and releasing potent ECFCs into the umbilical circulation, the fetus contributes to placental vascularization [8]. From this study it looks feasible that similar ECFCs assist in maternal perfusion of the human placenta, expanding uterine microvasculature by being actively involved in formation of de novo endothelium during pregnancy. From these results, it is noteworthy that fetal-derived endothelial cells were identified in abundance in myometrial arteries distal to the utero-placental bed, that is, the point of placental origin. In these distal sites, it could be envisaged that infiltrating ECFCs have a more classic role in vasculogenesis and vascular expansion. Nevertheless, their additional importance in vascular repair cannot be discounted, especially within the placenta-bed and more specifically proximal, transformed spiral arteries. Given the likelihood of re-endothelization of these arteries following trophoblast remodeling [29], it is tantalizing to consider that this restored endothelium is wholly recolonized and re-established by fetal ECFCs. Although this suggestion is hard to confirm, given the practicalities of obtaining placenta-bed biopsies and undoubted presence of contaminating trophoblast, this finding would place fetal-ECFCs on a par with invading trophoblasts with regard to human placentation, placing fundamental importance on their integrity for successful pregnancy.

As both adult and fetal ECFCs have vasculogenic capacities, and endothelial cells derived from EPCs are not subject to a maternal immune response [44], ECFCs of fetal or maternal origin may, in theory, concomitantly contribute to vascular expansion in the pregnant uterus. However, with fetal ECFCs superior to the adult in both their vasculogenic and proliferative capabilities [16, 54, 55], it is anticipated that contributions would favor fetal cells, particularly given the infrequency of adult ECFCs in the peripheral circulation (1 cell per 10 milliliter) [18]. Nevertheless, without substantive data, the relative contribution of maternal and fetal ECFCs within the pregnant uterus remains undecided.

For a number of obstetric complications, utero-placental irregularities are considered pathogenic cornerstones. Thus, within pre-eclampsia and IUGR, suboptimal conditions are often forewarned by sonographically defined elevations in uterine artery resistance. As a surrogate for downstream arboration and vessel distensibility, these uterine artery measurements arguably highlight suboptimal microvascularization. Given the abundance of fetal ECFCs in these affected microvessels, restricted numbers or function could constitute a realistic pathogenic phenomenon. In pre-eclampsia, vascular inflammation and maternal endothelial dysfunction have been linked to restricted maternal EPC numbers and proficiency [56], although phenotypic definition and quantification challenge this suggestion [14]. Whether these changes in EPCs (inclusive of ECFCs) precede the clinical condition is unknown. Two recent reports, highlighting a decrease in cord-blood EPCs in pre-eclamptic women with unclassified and severe disease [57, 58], certainly concur with a maternal dependence on these fetal cells. Our new finding links these observations, perhaps shifting importance away from circulating adult-derived EPCs in preeclampsia, to the far more efficient ECFCs of the fetus, residing within, but perhaps not restricted to, the maternal vascular uterus.

Within the fetal circulation, recent studies have associated aberrant EPC numbers and function with preterm birth and fetal growth restriction [13, 59]. These anomalies may impact upon placental vascular development, restricting nutrient supply. If these same abnormalities persist in chimeric ECFCs, then a concomitant effect on maternal vascular adaptations would be predicted, and indeed this notion is upheld with the clinical overlap between IUGR and early onset pre-eclampsia [60]. Moreover, given that vascular repair is a perquisite for cardiovascular health [61], it would also be predicted that fetal ECFCs that underperform in the fetus and maternal uterus in pregnancy could equally underperform in later life. Epidemiological evidence supports this, with both mothers and babies affected by IUGR and pre-eclampsia showing increased susceptibility to adult cardiovascular disease [4, 5].

## CONCLUSION

In summary, this is the first confirmation of fetal ECFC passage across the human placenta and first description of fetal cells maintaining active function following transmigration. Their frequency and relative activity in comparison to native cells would propose a profound role in uterine vascular expansion and optimal placentation, and speculations regarding their pathogenic role in pregnancy seem warranted. For description of this novel phenomenon, we propose the term functional feto-maternal macrochimerism. Their subsequent involvement in maternal autoimmune disease and long-term cardiovascular risk are likewise intriguing propositions.
